# Sustainable diets and long-term cardiovascular disease outcomes; insights from the 20-year follow-up ATTICA study (2002–2022)

**DOI:** 10.1007/s00394-026-04022-7

**Published:** 2026-06-19

**Authors:** Evangelia G. Sigala, Evangelia Damigou, Dimitrios Dalmyras, Christina Chrysohoou, Fotios Barkas, Christos Pitsavos, Costas Tsioufis, Demosthenes Panagiotakos

**Affiliations:** 1 Department of Nutrition and Dietetics, School of Health Sciences and Education, Harokopio University, Athens, 17671 Greece; 2 First Cardiology Clinic, Medical School, National and Kapodistrian University of Athens, Hippokration Hospital, Athens, 15772 Greece; 3 Department of Internal Medicine, Medical School, University of Ioannina, Ioannina, 45500 Greece

**Keywords:** Cardiovascular disease epidemiology, Risk assessment, Primary prevention, Dietary pattern, Sustainable diets

## Abstract

**Purpose:**

To identify habitual dietary patterns within a Mediterranean population-based cohort, examine their associations with cardiovascular disease (CVD) risk factors and outcomes over 20 years, and interpret them in sustainability terms.

**Methods:**

A total of 3,042 CVD-free adults from the ATTICA Study were enrolled in 2001–2002. Dietary intake was assessed using a validated food frequency questionnaire, and principal component analysis was utilized to derive *a posteriori* dietary patterns. Participants were followed for 20 years, with complete CVD data available for 1988 individuals. CVD incidence, lifetime risk, and disability-adjusted life years (DALYs) were calculated. Multivariate Cox proportional hazards, beta and gamma regression models examined associations between dietary patterns and CVD outcomes.

**Results:**

Three dietary patterns were identified, explaining 46.5% of variance in consumption: a plant-based, sustainable pattern; a Western pattern rich in animal-sourced and processed foods; and a high-calorie, low-white-meat pattern. Higher adherence to the plant-based pattern was associated with a 26% (HR: 0.74, 95% CI 0.55–0.99) lower 20-year CVD risk. The high-calorie, low-white-meat pattern was linked to increased lifetime risk (β = 0.066, *p* < 0.001) and DALYs (β = 0.13, *p* = 0.009). No significant associations were observed for the Western pattern after adjustments.

**Conclusions:**

A plant-based dietary pattern was protective against long-term CVD outcomes, while a high-calorie, low-white-meat pattern was detrimental. These findings underscore the importance of promoting culturally acceptable, sustainable dietary patterns to reduce CVD risk and support environmental sustainability.

**Supplementary Information:**

The online version contains supplementary material available at 10.1007/s00394-026-04022-7.

## Introduction

Between 2010 and 2021, the age-standardized cardiovascular disease (CVD) rates per 100,000 population exhibited slight-to-modest declines of 1.5% in incidence, 14.5% in mortality, and 14.0% in disability-adjusted life years (DALYs) globally [1]. These trends largely reflect advancements in elucidating underlying pathophysiological mechanisms, the implementation of preventive measures, and improvements in treatments. Nevertheless, driven by population growth and demographic aging, crude epidemiological indices rose substantially over the same period, with incident CVD cases increasing by 30.5%, deaths by 18.5%, and DALYs by 14.3% [1]. Projections under a business-as-usual scenario remain concerning, forecasting that from 2022 to 2050, CVD-related fatalities will surge by 40.8% and DALYs by 19.7% [2], thereby underscoring mounting strains on healthcare systems and the imperative for primordial and primary prevention, including adoption of healthier diets [3, 4]. Poor dietary habits represent a principal modifiable risk factor contributing to this growing burden. In 2021, suboptimal consumption patterns characterized by insufficient intake of nutrient-dense foods were responsible for approximately 6.6 million CVD deaths and 1,420 DALYs per 100,000 individuals [5]. Hence, from a food system perspective, this highlights a critical need for current food systems, i.e., the “*backbone of global diets*” [6], to ensure equitable access to diets and food environments that promote health [7].

Concurrently, unsustainable consumption and production patterns within food systems intensify environmental degradation, thereby compromising their capacity to deliver nutritious diets [7, 8]. The global food system accounts for approximately 21–37% of anthropogenic greenhouse gas (GHG) emissions, predominantly from livestock and crop production; land-use changes such as deforestation; and inefficiencies in food supply chains, including food losses and waste [8]. Additionally, food systems exert substantial pressure on freshwater resources, with an estimated annual blue water footprint of 1810 km³; drive extensive land-use change, occupying nearly 12.6 million km² of cropland; contribute to biodiversity loss; and exacerbate eutrophication in aquatic and terrestrial ecosystems through excessive nitrogen and phosphorus fertilizers application [9]. Meanwhile, a reinforcing vicious cycle exists wherein climate change further impairs food systems; for example, by reducing agricultural productivity, especially in warming and drying regions like the Mediterranean peninsula, altering phenological seasons, and decreasing the nutrient density of staple crops [8]. These environmental challenges, combined with social and economic inequities, exacerbate food insecurity and adversely affect human health [7, 8]. Future trajectories of population growth, urbanization, affluence in developing societal strata, and rising demand for resource-intensive foods, together with climate change and non-climate stressors such as economic downturns and geopolitical instabilities, are anticipated to put added strain on Earth’s boundaries [8, 9]. Consequently, these factors will likely undermine further food systems’ resilience and stability, limit their capacity to supply safe, sufficient, and nutritious food, and amplify human vulnerability [8].

In light of these multifaceted challenges, a paradigm shift toward the adoption of *healthy and sustainable dietary patterns* has been advocated as a pivotal demand-side mitigation strategy to improve health outcomes and curb environmental deterioration [8]. These diets emphasize diverse plant-based foods and limit animal-sourced food products, particularly those from resource-intensive, high-GHG emission systems, while prioritizing seasonal and locally produced foods [7, 8, 10, 11]. Such patterns can enhance individual health and well-being, reduce environmental footprint, and ensure affordability, accessibility, and equity, all while maintaining safety and cultural acceptability across generations. Thus, this holistic concept harmonizes health objectives with the environmental, economic, and social pillars of sustainability.

Over the past decades, nutritional epidemiology has transitioned from focusing on isolated nutrients, foods, or food groups to analyzing overall dietary patterns [12]. This holistic perspective acknowledges the complexity of dietary intake, wherein individuals consume a combination of nutrients and bioactive compounds embedded within food matrices, and the consumption of these foods in specific amounts, combinations, proportions, and frequencies exerts synergistic or competing effects [13]. Two principal methodological approaches are employed to identify dietary patterns [12]: a priori or *hypothesis-driven methods*, in which dietary indices or scoring systems are constructed based on established scientific evidence to quantify adherence to dietary guidelines, recommendations, or traditional diets (e.g., the Mediterranean diet [14]); and *a posteriori* or *data-driven methods*, in which dietary patterns are empirically derived from dietary intake datasets using multivariate analyses.

Although many investigators have applied a priori indices to assess the relationship between sustainable dietary patterns and CVD-related outcomes, such as scores based on the EAT-Lancet dietary pattern (EAT-LDP) [15–34] recommended by the Lancet Commission [10] and the Sustainable Diet Index (SDI) [35], such approaches often fail to capture actual dietary habits within populations [36]. Hence, this study aims to identify *a posteriori* dietary patterns that reflect habitual dietary intake within a Mediterranean cohort (i.e., the 20-year ATTICA Study), interpret these patterns in terms of health and sustainability, and examine their associations with CVD risk factors, fatal and non-fatal CVD incidence, remaining lifetime CVD risk, and attributed DALYs.

## Methods

### Study design, recruitment, and eligibility

The scope, design, and procedures of the ATTICA Study have been detailed previously [37–40]. Briefly, the ATTICA Study is an observational study featuring a prospective design over a 20-year period. To achieve a representative sample of the underlying population, multistage stratified random sampling was conducted across 27 municipalities of Attica, Greece (78% urban, 22% suburban areas), with strata defined by age (five brackets) and biological sex (male/female) distributions from the 2001 national Population-Housing Census. One subject per household was invited for participation. Eligibility criteria included no prior history of CVD, cancer, or other inflammatory medical conditions, nor any surgical procedure within the preceding week. Of the 4,056 apparently healthy adults invited, 3042 consented and were recruited at baseline in 2001–2002 (75% participation rate) [39].

Three follow-up examinations were conducted at 5 years (69% participation rate) [40], 10 years (85% participation rate) [38], and 20 years (71% participation rate) [37] after the baseline. During each follow-up examination, surviving volunteers were contacted by telephone to schedule in-person assessments.

For participants who were deceased prior to a scheduled follow-up, cause and date of death were ascertained from family members and official death registration certificates. At the long-term (20-year) assessment, 1988 out of the 2169 volunteers had complete data on CVD endpoints [37]. No differences were observed in the age–sex distribution between the 20-year follow-up sample with complete CVD data and the baseline cohort (p*s* > 0.80) [37]. However, participants lost to follow-up exhibited a less favorable socioeconomic profile at baseline, as reflected by fewer schooling years (*p =* 0.004) and a higher prevalence of adverse financial status (*p =* 0.037).

### Data collection at each examination

All assessments were conducted by trained healthcare professionals following ATTICA Study protocols [37–40]. Structured questionnaires captured socio-demographic characteristics (e.g., date of birth, sex, occupation, mean annual income, educational attainment, marital status, number of offspring), lifestyle behaviors (diet, smoking, physical activity, and leisure-time activities), family history of CVD, and personal medical and pharmacological history. Physical examinations (i.e., arterial blood pressure and anthropometric measurements) and morning blood sampling for laboratory analyses were performed only at the baseline. Additionally, participants not meeting the eligibility criteria were excluded from participation following a detailed clinical assessment.

### Ascertainment of CVD cases

The primary endpoints comprised fatal and non-fatal CVD episodes, classified according to the World Health Organization (WHO)-International Classification of Diseases, 9th and 10th revisions [37–40]. Non-CVD attributable mortality was also systematically documented to enable adjustment for competing risks in lifetime risk estimations.

### Assessment of clinical, laboratory, anthropometric, and lifestyle characteristics

The medical history assessment focused on ascertaining prevalent hypertension, hypercholesterolemia, and type 2 diabetes mellitus (hereafter termed diabetes mellitus) [39]. Hypertension was diagnosed by use of antihypertensive pharmacotherapy or systolic and/or diastolic blood pressure > 140/90 mmHg. Hypercholesterolemia was defined by use of lipid-lowering medications or total serum cholesterol > 200 mg/dL. Diabetes mellitus was confirmed by the use of relevant medication or fasting plasma glucose levels ≥ 126 mg/dL. All laboratory assays (e.g., lipid profile, hematological indices, markers of glucose metabolism, hepatic and renal function, as well as inflammatory and coagulation biomarkers) were performed at a single certified reference laboratory complying with WHO quality standards.

Anthropometric measurements encompassed body weight (kg), height (cm), waist circumference (WC; cm), and hip circumference (cm), obtained utilizing standardized procedures and calibrated equipment (digital scale, stadiometer, non-elastic tape) [39]. Body mass index (BMI; kg/m^2^) was determined as weight divided by height squared and categorized per WHO criteria: obesity (≥ 30.0 kg/m^2^), overweight (25.0–29.9 kg/m^2^), normal (18.5–24.9 kg/m^2^), and underweight (< 18.5 kg/m^2^) [41]. Sex-specific central obesity cut-offs for WC were ≥ 88 cm and ≥ 102 cm for females and males, respectively [42]. Waist-to-height (WHtR; cut-off ≥ 0.50 for both sexes) [43] and waist-to-hip (WHR; thresholds ≥ 0.80 in females and ≥ 0.95 in males) [44] ratios were also computed.

Smoking status was classified as ever smokers (current or former) versus never smokers [39]. Current smokers reported consuming ≥ 1 cigarette/day or having ceased smoking within the preceding year; former smokers had abstained for > 1 year; never smokers reported no history of tobacco use. Cumulative smoking exposure was quantified in pack-years, calculated as the number of packs (20 cigarettes/pack) smoked per day multiplied by years of smoking. Physical activity was assessed using the validated Greek version of the Short Form of the International Physical Activity Questionnaire (IPAQ-SF) [45], with activity episodes defined as recreational activities sustained ≥ 10 min. Sedentary lifestyle was defined as the absence of any reported episode, whereas any level of participation beyond this threshold was considered active and subsequently categorized by intensity as light, moderate, or vigorous, based on per-minute caloric expenditure.

### Dietary intake assessment

Usual dietary intake was evaluated by trained dietitians via the validated semi-quantitative EPIC food frequency questionnaire (FFQ), which consists of 156 food and beverage items representative of the Greek diet, including: vegetables and leafy greens (e.g., iceberg, spinach, beetroots, eggplants), fruits (e.g., apples, oranges, bananas, melon), grain groups (refined and whole-grain bread, crackers, pasta, rice), legumes (e.g., lentils, beans), nuts, potatoes (fried, baked, boiled, mashed), seafood and fish (categorized by size), various meat types (beef, pork, ruminant meat, poultry) and their products, eggs, dairy by fat content (milk, yogurt, cheese, creams), sweets and confectionery (e.g., cakes, chocolate), added fat (olive oil, other seed oils, margarine, butter), tea, coffee, and alcoholic beverages (e.g., wine, beers, distilled spirits) [46]. Composite dishes were disaggregated into ingredients and allocated to the corresponding food groups. Participants were instructed to report the frequency of consumption for each item in daily or weekly servings, reflecting their habitual dietary intake over the preceding month. Assessments were conducted continuously over 12 months to capture seasonal variation. Portion-size photographs were provided to enhance estimation accuracy. Alcohol consumption was standardized to 100 mL wineglass equivalents, each containing 12 g of ethanol.

### Identification of *a posteriori* dietary patterns and other statistical procedures

Dietary patterns were empirically derived using principal components analysis (PCA), a multivariate statistical technique widely used in the field of nutritional epidemiology to reduce the dimensionality of dietary intake datasets by extracting principal components that explain the greatest proportion of total variance [12]. Prior to PCA, all food group variables and energy intake adjusted to basal metabolic rate (BMR) (*see* Table 1) were standardized to z-scores. Added fat was excluded from analysis due to the limited data variability, as nearly all volunteers reported daily olive oil consumption. Data adequacy for PCA was confirmed by a Kaiser-Meyer-Olkin measure of 0.756, a significant Bartlett’s test of sphericity (*p* < 0.001), and a correlation matrix determinant of 0.181. PCA was applied to the correlation matrix of z-standardized variables. To enhance interpretability and ensure statistical independence among components reflecting the dietary patterns, an orthogonal Varimax rotation was performed. The number of retained components was determined by the Kaiser criterion (eigenvalues > 1.0), visual inspection of the Cattell scree plot, and the interpretability of the constructed patterns. Variables with factor loadings of ≥|0.25| were deemed significant contributors to each component.

Ordinal and nominal variables are summarized as relative frequencies, and non-Gaussian numerical variables as medians with interquartile ranges. Crude cumulative CVD incidence and mortality proportions were calculated as new cases divided by the number of adults enrolled at follow-up. Remaining lifetime risk of developing CVD was calculated from enrollment to the baseline age of 80, as data beyond this age were sparse. Lifetime risk by tertiles of *a posteriori* dietary patterns was derived using a modified life-table Kaplan-Meier method with competing risk adjustments for non-CVD mortality to prevent overestimation [47]. CVD burden was quantified in DALYs as the combined total of years lived with CVD-related disability and years of life lost due to premature CVD mortality. To compare tertiles, the chi-square test was used for categorical variables, the non-parametric Kruskal-Wallis test for skewed continuous variables, and the log-rank test for cumulative fatal and non-fatal incidence. The association between adherence to the data-driven dietary patterns and 20-year incidence was assessed using nested Cox proportional hazards regression models, with hazard ratios (HR) and 95% confidence intervals (95% CI) calculated following hierarchical adjustments for blocks of potential CVD predictors, entered in order of descending effect size. Additionally, beta and gamma regression models were employed to evaluate the multi-adjusted associations between adherence to the extracted patterns and CVD lifetime risk and DALYs, respectively. Results are presented as β-coefficients (β) and standard errors (SE), with exponentiated β interpreted as effect measures. All *p-*values were two-sided tests and subsequently contrasted at the 0.05 significance level. Statistical analyses were performed in STATA version 18 (STATA Corp., College Station, Texas, USA).

## Results

## *A posteriori* dietary patterns

**Fig. 1:**
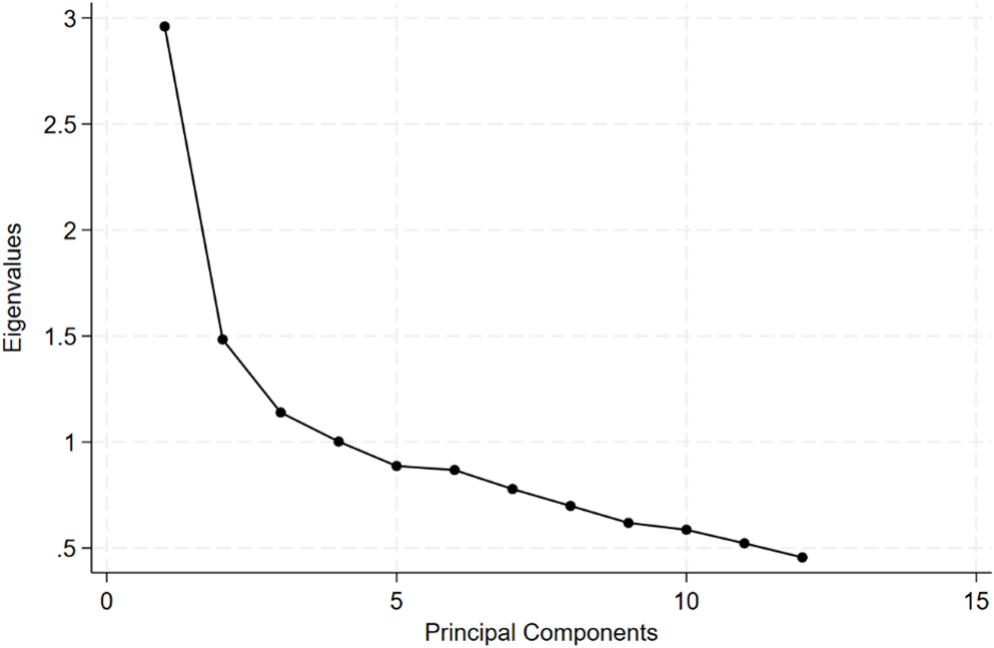
Eigenvalue scree plot by principal component number, used alongside the Kaiser criterion and interpretability to guide component retention

These analyses were conducted on the baseline cohort of 3,042 participants (females: 50.2%, 45 ± 14 years old; males: 49.8%, 46 ± 13 years old) with complete dietary intake data. Three principal components were identified via PCA (Fig. 1), collectively explaining 46.5% of the total variance in baseline dietary intake (Table 1). The first component reflected a plant-based, sustainable dietary pattern, with greater consumption of vegetables and leafy greens, fruits, foods abundant in plant-based protein (legumes and nuts), grains, fish and seafood, and dairy products. The second component was marked by increased consumption of red meat and products, potatoes, sweets and confectionery, and eggs, indicative of a Western dietary pattern rich in animal-based and processed foods. The third component represented a pattern characterized by elevated energy intake relative to BMR and low white meat consumption. To explore potential non-linear effects in subsequent analyses, participants were classified into tertiles based on their factor scores for each identified dietary pattern (T1: lowest adherence, T2: moderate adherence, T3: highest adherence).

**Table 1:** Rotated component score coefficient matrix derived from PCA of the baseline food consumption among ATTICA Study participants with complete dietary assessment (*n* = 3042)

Food groups	Components		
	1	2	3
Vegetables and leafy greens	**0.5056**	− 0.1399	− 0.0996
Fruits	**0.4518**	− 0.0478	− 0.0194
Grain group	**0.3724**	0.0637	− 0.1227
Plant-based protein foods	**0.3895**	0.0797	0.1742
Potatoes	− 0.0439	**0.5437**	− 0.0267
Red meat and products	− 0.0931	**0.5695**	− 0.1575
White meat and products	− 0.0156	0.1715	**− 0.6744**
Eggs	0.0877	**0.3420**	0.1435
Dairy products	**0.2580**	0.2113	− 0.0291
Fish and seafood	**0.3725**	− 0.0392	0.0399
Sweets and confectionery	0.1478	**0.3737**	0.2117
Energy intake adjusted to BMR	− 0.0646	0.1248	**0.6294**
*Variance explained*,* %*	*20.6*	*16.2*	*9.7*

Bold values indicate factor loadings ≥|0.25|, considered significant contributors to each component. Italic values indicate the variance explained by each component. BMR: basal metabolic rate

### Baseline CVD risk factors by tertiles of dietary patterns

Participants’ baseline characteristics across tertiles of each empirically derived dietary pattern are detailed in Table 2. In summary, volunteers in the highest tertile of the plant-based, sustainable dietary pattern were older and exhibited an overall more heart-healthy lifestyle compared to those in the lower tertiles. In contrast, greater adherence to a Western pattern rich in animal-based and processed foods was mainly observed among younger participants and males. Notably, subjects in the second and third tertiles of this pattern had lower cumulative smoking exposure and a reduced prevalence of hypercholesterolemia, while male participants exhibited significantly lower WHtR and WHR values than those in the first tertile. These associations are likely influenced by the unadjusted nature of the analysis and the overrepresentation of younger individuals among higher adherence tertiles, with 70.2% and 77.7% of participants in the second and third tertiles, respectively, being under 45 years of age. Conversely, higher adherence to a pattern characterized by caloric intake exceeding metabolic needs and low white meat consumption was linked with younger age, increased prevalence of overweight and obesity, and less favorable anthropometric profiles.

**Table 2:** Baseline socio-demographic, clinical, anthropometric, and lifestyle characteristics stratified by tertiles of dietary patterns among CVD-free participants of the ATTICA Study (*n* = 3042)

	Plant-based, sustainable dietary pattern				Western animal-based, processed food pattern				High-calorie, low-white-meat dietary pattern			
Variables	T1	T2	T3	*p* for trend^a^	T1	T2	T3	*p* for trend^a^	T1	T2	T3	*p* for trend^a^
Baseline age, *years*	39 (16)	41 (17)	42 (18)	*0.041*	44 (15)	40 (15)	35 (17)	< *0.001*	42 (16)	40 (16)	38 (18)	*0.002*
Sex, *% males*	54.3	56.3	50.6	0.328	47.8	51.8	61.9	*0.001*	52.2	54.5	54.5	0.798
Socio-economic status ^b^, *%*				*0.005*				0.287				0.662
Low class	8.5	8.6	10.5		10.0	7.3	10.1		11.0	8.7	7.7	
Middle class	58.5	43.8	50.7		48.8	49.7	54.6		49.0	51.0	53.2	
High class	33.0	47.6	38.8		41.3	43.0	35.4		40.0	40.3	39.2	
Hypertension, *%*	25.8	26.0	30.0	0.392	27.5	29.7	24.5	0.332	25.1	27.2	29.6	0.442
Hypercholesterolemia, *%*	28.8	34.8	28.9	0.151	40.1	25.9	26.5	< *0.001*	27.6	32.4	32.4	0.291
Diabetes mellitus, *%*	5.9	4.2	3.3	0.235	5.9	4.5	3.0	0.178	3.9	3.6	6.0	0.264
Overweight and/or obesity, *%*	53.0	52.7	54.2	0.920	57.4	50.3	52.1	0.155	46.9	55.2	57.7	*0.013*
Increased WC, *%*	43.9	46.1	51.5	0.129	51.3	44.9	45.2	0.172	40.7	51.2	49.7	*0.012*
BMI, *kg/m*^*2*^	25.3 (6.2)	25.3 (5.7)	25.3 (5.3)	0.997	25.6 (5.9)	25.0 (5.4)	25.2 (5.6)	*0.048*	24.7 (4.8)	25.4 (5.9)	25.9 (6.4)	*0.001*
WC, *cm*	89 (23)	88 (22)	89 (23)	0.888	90 (25)	88 (21)	90 (22)	0.419	87 (21)	89 (22)	90 (23)	*0.010*
Females	76 (16)	76 (16)	78 (15)	0.149	78 (18)	78 (13)	75 (16)	0.061	76 (12)	78 (14)	78 (19)	0.126
Males	95 (14)	96 (16)	97 (15)	0.325	98 (17)	95 (14)	95 (14)	0.067	95 (13)	96 (16)	97 (16)	0.053
WHtR	0.52 (0.11)	0.51 (0.11)	0.51 (0.11)	0.827	0.53 (0.12)	0.51 (0.10)	0.51 (0.11)	0.122	0.51 (0.11)	0.52 (0.11)	0.52 (0.11)	*0.045*
Females	0.47 (0.11)	0.46 (0.09)	0.47 (0.09)	0.194	0.47 (0.12)	0.47 (0.09)	0.46 (0.10)	0.075	0.46 (0.10)	0.47 (0.09)	0.48 (0.12)	0.145
Males	0.54 (0.08)	0.55 (0.09)	0.55 (0.09)	0.401	0.56 (0.09)	0.54 (0.08)	0.53 (0.09)	*0.006*	0.54 (0.08)	0.54 (0.08)	0.55 (0.09)	0.220
WHR	0.87 (0.14)	0.86 (0.16)	0.84 (0.15)	0.386	0.86 (0.16)	0.85 (0.15)	0.86 (0.13)	0.783	0.85 (0.15)	0.86 (0.15)	0.87 (0.15)	0.233
Females	0.78 (0.10)	0.77 (0.07)	0.79 (0.08)	0.136	0.78 (0.09)	0.78 (0.08)	0.78 (0.07)	0.456	0.77 (0.07)	0.78 (0.08)	0.78 (0.09)	0.192
Males	0.92 (0.08)	0.92 (0.09)	0.92 (0.08)	0.051	0.93 (0.09)	0.92 (0.09)	0.90 (0.09)	*0.003*	0.91 (0.09)	0.92 (0.09)	0.92 (0.09)	0.735
Past/ current smoking, *%*	51.9	43.8	40.5	*0.009*	45.4	45.2	45.5	0.997	43.9	45.8	46.4	0.791
Smoking exposure, *pack-years*	308 (605)	330 (445)	300 (460)	0.058	390 (500)	285 (425)	255 (475)	*0.002*	360 (515)	300 (480)	300 (420)	0.133
Sedentary lifestyle, *%*	65.3	53.9	47.9	< *0.001*	54.3	54.8	58.0	0.569	53.1	61.0	53.0	0.056

Continuous variables are expressed as medians (interquartile ranges), and categorical data as relative frequencies (%). ^a^ Between-group differences were evaluated via the Kruskal-Wallis test and the chi-square test for numerical and categorical variables, respectively. ^b^ The socio-economic status was assessed as an aggregate estimate of educational attainment and mean annual income over the past three years, then classified as follows: low class (income ≤€8,000 and educational level < 14 years; income ≤€8,000 or €8,001–10,000 and educational level < 9 years), high class (income >€20,000 and educational level 10–14 years; income €10,001–20,000 or >€20,000 and educational level ≥ 15 years), and middle class (all other combinations)

BMI: body mass index, Ti: i^th^ tertile, WC: waist circumference, WHR: waist-to-hip ratio, WHtR: waist-to-height ratio

### Crude analysis of CVD outcomes by tertiles of dietary patterns

Among the 1988 participants with complete 20-year CVD data, 36.0% (females: 320/1000; males: 400/1000; *p* < 0.001) experienced a fatal or non-fatal CVD episode. Fatal events accounted for 4.8% of total events (females: 18/1000; males: 73/1000; *p* < 0.001). Key epidemiological indices according to the tertiles of each data-driven dietary pattern are presented in Table 3. Lower adherence to a plant-based, sustainable pattern and higher adherence to a high-calorie, low-white-meat pattern were associated with higher remaining lifetime risk from the respective age index. As for the Western pattern rich in animal-based and processed foods, higher adherence was linked to earlier CVD onset, yet the 10- and 20-year cumulative CVD incidence estimates were paradoxically lower; this finding may be attributable to residual confounding by unadjusted predisposing and protective CVD factors in this univariate analysis.

**Table 3:** Crude 10- and 20-year cumulative CVD incidence, mortality, lifetime risk estimates, and DALYs, stratified by tertiles of the dietary patterns among participants of the ATTICA Study (*n* = 1988)

Epidemiological indices	Plant-based, sustainable dietary pattern				Western animal-based, processed food pattern				High-calorie, low-white-meat dietary pattern			
	T1	T2	T3	*p* for trend ^a^	T1	T2	T3	*p* for trend ^a^	T1	T2	T3	*p* for trend ^a^
Age at CVD event, *years*	58 (15)	62 (12)	60 (14)	0.450	61 (11)	62 (9)	55 (18)	*0.035*	60 (11)	59 (11)	61 (19)	0.983
10-year CVD incidence, *%*	12.1	8.5	12.5	0.208	16.4	8.0	8.9	*0.007*	11.7	9.8	11.8	0.721
≤ 35 years old	2.4	5.5	6.9		6.0	2.6	6.1		6.1	1.3	6.9	
35–45 years old	7.7	1.4	3.9		8.6	2.4	1.6		3.8	4.5	5.2	
45–55 years old	31.9	14.9	21.7		27.3	12.9	25.7		23.4	24.5	17.5	
55–65 years old	26.7	36.4	40.9		31.8	50.0	25.0		23.8	33.3	58.3	
> 65 years old	–	100.0	100.0		100.0	–	100.0		100.0	100.0	100.0	
20-year CVD incidence, *%*	22.0	24.9	25.0	0.602	33.6	20.9	17.3	*< 0.001*	27.2	20.6	24.2	0.188
≤ 35 years old	3.8	5.5	8.1		8.0	2.5	7.0		7.5	1.3	7.9	
35–45 years old	8.9	1.4	5.2		9.9	3.5	1.6		3.9	6.7	5.2	
45–55 years old	57.5	60.3	54.1		58.4	52.4	63.9		60.0	50.9	60.3	
55–65 years old	90.9	100.0	87.5		95.3	100.0	75.0		88.2	100.0	100.0	
> 65 years old	100.0	100.0	100.0		–	–	–		–	–	–	
20-year CVD mortality, *%*	1.2	0.8	1.1	0.876	1.2	0.8	1.2	0.862	1.6	0.8	0.8	0.580
Lifetime risk, *%* (95% CI)												
40–50 years old	71.1 (68.7, 73.6)	69.5 (67.1, 71.9)	70.8 (68.3, 73.3)	0.769	70.6 (68.4, 72.9)	69.9 (67.6, 72.3)	71.1 (68.2, 74.0)	0.711	68.1 (65.9, 70.4)	71.3 (69.0, 73.5)	72.6 (69.7, 75.5)	*0.022*
50–60 years old	67.9 (64.7, 71.1)	65.8 (63.1, 68.4)	62.9 (60.0, 65.4)	*0.048*	66.7 (64.3, 69.2)	63.9 (60.9, 66.9)	63.9 (60.1, 67.6)	0.174	63.3 (60.9, 65.7)	63.9 (60.7, 67.0)	68.9 (65.6, 72.1)	*0.020*
60–70 years old	69.0 (66.7, 71,4)	61.5 (54.6, 68.4)	66.6 (60.8, 72.5)	0.433	67.9 (64.6, 71.3)	62.7 (55.0, 70.4)	65.1 (57.1, 73.2)	0.495	61.9 (54.6, 69.2)	71.3 (68.2, 74.4)	61.6 (57.3, 65.9)	*0.013*
DALYs (95% CI)	16.5 (13.4, 19.6)	13.4 (10.7, 16.0)	17.0 (13.9, 20.0)	0.151	14.8 (12.5, 17.1)	13.7 (10.9, 16.7)	19.5 (15.5, 23.5)	0.068	14.6 (11.7, 17.4)	14.9 (12.1, 17.6)	17.5 (14.4, 20.6)	0.315

Continuous variables are expressed as medians (interquartile ranges), and categorical data as relative frequencies (%). ^a^ *p*-values were derived using the log-rank test for fatal and non-fatal incidence and the Kruskal-Wallis test for continuous variables

*Abbreviations*: DALYs: disability-adjusted life years, Ti: i^th^ tertile, 95% CI: 95% confidence interval

### Multivariate analysis of CVD epidemiological indices by dietary pattern adherence

Due to potential bias in crude analyses, multivariable-adjusted models were employed to elucidate the relationships between dietary habits and CVD outcomes and to alleviate residual confounding. In the fully adjusted Cox proportional hazards model, each 1 standard deviation (SD) increase in adherence to the plant-based, sustainable dietary pattern was associated with a 26% (HR: 0.74, 95% CI 0.55–0.99) reduction in 20-year CVD risk, revealing a significant cardioprotective effect (Table 4). In the tertile-based analysis (Supplementary Table S1, online resource), participants in the highest tertile of adherence to the plant-based, sustainable dietary pattern exhibited a 61% lower odds of 20-year CVD incidence compared with those in the lowest tertile (T3 vs. T1: HR: 0.39, 95% CI: 0.15–0.98), after full adjustment for demographic, socioeconomic, clinical, and lifestyle covariates, with a significant linear trend across ascending tertiles (*p* for trend = 0.047).

Results from the fully controlled beta regression model showed that each 1 SD increment in adherence to a high-calorie, low‐white‐meat pattern was correlated with a 7% (β ± SE: 0.066 ± 0.018, *p* < 0.001) increase in the odds of having greater lifetime CVD risk (Table 5). Additionally, engagement in physical activity was also cardioprophylactic (β ± SE: -0.12 ± 0.01, *p* = 0.002), whereas smoking increased lifetime CVD risk (β ± SE: 0.52 ± 0.04, *p* < 0.001). Similarly, in the gamma regression model, a 1-SD increase in adherence to a pattern high in calories and low in white meat was independently associated with a 14% (β ± SE: 0.13 ± 0.05, *p* = 0.009) higher CVD-attributable DALYs. In the tertile-based analysis, higher adherence to the high-calorie, low-white-meat dietary pattern was also associated with greater lifetime risk and higher DALYs, with significant linear trends across ascending tertiles (Supplementary Table S2, online resource).

To further mitigate concerns of reverse causality, whereby subclinical disease at baseline could have influenced dietary reporting and/or prompted early dietary modifications, we repeated the fully adjusted models after excluding fatal and non-fatal CVD events occurring within the first five years of follow-up. The direction and magnitude of the associations between adherence to the extracted dietary patterns and 20-year CVD outcomes remained materially unchanged.

**Table 4:** Results from nested Cox proportional hazards models assessing the relationship between adherence to the three orthogonal dietary patterns and 20-year CVD incidence among participants of the ATTICA Study (*n* = 1988)

Plant-based, sustainable dietary pattern, per 1 SD	Model 1	Model 2	Model 3	Model 4
	1.07 (0.88, 1.30)	0.81 (0.62, 1.06)	0.77 (0.58, 1.03)	0.74 (0.55, 0.99) ^*^
Western animal-based, processed food pattern, *per 1 SD*	0.73 (0.59, 0.92) ^*^	1.07 (0.77, 1.44)	1.13 (0.82, 1.55)	1.12 (0.81, 1.54)
High-calorie, low-white-meat dietary pattern, *per 1 SD*	0.74 (0.56, 0.97) ^*^	0.92 (0.66, 1.30)	0.88 (0.61, 1.27)	0.87 (0.61, 1.26)
Age, *per 1 year*		1.23 (1.17, 1.30) ^***^	1.22 (1.15, 1.29) ^***^	1.22 (1.15, 1.30) ^***^
Sex, *ref: females*		2.89 (1.39, 6.01) ^**^	2.55 (1.05, 6.17) ^*^	2.58 (1.06, 6.27) ^*^
Socio-economic status, *ref: low class*				
Middle class		0.94 (0.29, 3.06)	1.05 (0.30, 3.68)	0.98 (0.29, 3.47)
High class		0.71 (0.21, 2.38)	0.70 (0.19, 2.56)	0.58 (0.26, 2.21)
Hypertension, *ref: normal*			1.56 (0.74, 3.31)	1.57 (0.73, 3.38)
Hypercholesterolemia, *ref: normal*			3.22 (1.52, 6.83) ^**^	3.31 (1.53, 20.0) ^*^
Diabetes mellitus, *ref: normal*			3.97 (0.79, 20.0)	4.63 (0.84, 25.5)
WHR, *per 1 unit*			0.06 (0.01, 3.88)	0.10 (0.01, 6.37)
Smoking exposure, *per 1 pack-year*				1.00 (0.99, 1.01)
Sedentary lifestyle status, *ref: sedentary*				1.62 (0.75, 3.49)
*p* of the omnibus test for the model	*0.004*	*< 0.001*	*< 0.001*	*< 0.001*
*p* of the likelihood ratio test	*0.004*	*< 0.001*	*0.003*	*0.238*

Results are presented as HR (95% CI)

Model 1: Plant-based, sustainable dietary pattern + Western animal-based, processed food pattern + High-calorie, low-white-meat dietary pattern

Model 2: Model 1 + Age + Sex + Socio-economic status

Model 3: Model 2 + Hypertension + Hypercholesterolemia + Diabetes mellitus + WHR

Model 4: Model 3 + Smoking exposure + Sedentary lifestyle status

^***^ *p* < 0.001, ^**^ *p* < 0.01, ^*^ *p* < 0.05

HR: hazards ratio, SD: standard deviations, 95% CI: 95% confidence interval

**Table 5:** Results from statistical models assessing the relationship between adherence to the three orthogonal dietary patterns and CVD-related lifetime risk and DALYs among participants of the ATTICA Study (*n* = 1,988)

	Lifetime risk	DALYs
Plant-based, sustainable dietary pattern, per 1 SD	0.001 ± 0.013	− 0.04 ± 0.05
Western animal-based, processed food pattern, *per 1 SD*	− 0.030 ± 0.016	0.01 ± 0.05
High-calorie, low-white-meat dietary pattern, *per 1 SD*	0.066 ± 0.018 ^***^	0.13 ± 0.05 ^**^
Age, *per 1 year*	0.009 ± 0.002 ^***^	− 0.06 ± 0.01^***^
Sex, *ref: females*	0.30 ± 0.05 ^***^	− 0.22 ± 0.15
Socio-economic status, *ref: low class*		
Middle class	0.07 ± 0.07	− 0.10 ± 0.15
High class	0.06 ± 0.07	− 0.11 ± 0.17
Hypertension, *ref: normal*	0.28 ± 0.05 ^***^	− 0.02 ± 0.10
Hypercholesterolemia, *ref: normal*	0.26 ± 0.05 ^***^	0.12 ± 0.11
Diabetes mellitus, *ref: normal*	0.33 ± 0.12 ^**^	0.18 ± 0.013
WHR, *per 1 unit*	0.80 ± 0.27 ^**^	− 0.45 ± 0.76
Smoking, *ref: non-smokers*	0.52 ± 0.04 ^***^	0.09 ± 0.11
Sedentary lifestyle status, *ref: sedentary*	− 0.12 ± 0.01 ^**^	− 0.08 ± 0.10

Results are presented as β ± SE

^***^ *p* < 0.001, ^**^ *p* < 0.01, ^*^ *p* < 0.05

DALYs: disability-adjusted life years, SD: standard deviations, SE: standard errors

## Discussion

Despite substantial research linking diet and CVD prevention, the nexus between actual socio-culturally determined dietary habits, long-term CVD outcomes, and sustainability remains understudied. This study aimed to address this gap by identifying *a posteriori* dietary patterns and assessing their associations with CVD outcomes using data from the ATTICA Study, a Mediterranean cohort followed for over two decades. Although data-driven analyses reflect actual consumption habits regardless of their healthfulness and thus may be less predictive of health outcomes than a priori patterns [36], our analysis yielded several new insights into the intricate interplay between dietary habits and CVD, integrating a sustainability perspective.

### Summary of findings

In this work, three data-driven dietary patterns were extracted, collectively explaining 46.5% of the total variance in dietary intake, slightly above that reported in other Mediterranean cohorts (i.e., median: 45.5%, range: 6.6–82.0%) [36]. The first principal component, a plant-based dietary pattern akin to the traditional Mediterranean diet, was characterized by higher consumption of vegetables and leafy greens, fruits, plant-sourced protein foods (legumes and nuts), grains, fish and seafood, and products, and accounted for nearly one-fifth of data variance. Importantly, this pattern emerged as the sole lifestyle factor linked to a 26% reduction in 20-year CVD risk after adjustment for a set of potential confounders. This finding was further supported by the tertile-based analysis. The second pattern featured greater consumption of red meat and products, potatoes, sweets and confectionery, and eggs, and explained 16.2% of the variance. Nevertheless, this pattern showed no significant association with CVD epidemiological indices after full adjustments, possibly due to the overrepresentation of younger individuals among those with greater adherence. Specifically, given that age is one of the strongest determinants of CVD risk, adjustment for age may have attenuated the initially observed associations, suggesting that the crude relationship was, at least in part, attributable to differences in baseline risk profile rather than reflecting an independent effect of the dietary pattern itself. Conversely, the third component, a calorie-rich pattern exceeding basal metabolic requirements coupled with low white meat intake, explained 9.7% of variance and was independently correlated with an elevated lifetime CVD risk and increased CVD burden expressed in DALYs. These results remained unaltered after excluding those who experienced CVD events up to the 5-year follow-up, supporting the robustness of the observed associations to potential reverse causality.

### *A posteriori* dietary patterns interpreted through the lens of healthiness and sustainability

The first principal component, labeled as “plant-based, sustainable,” aligns with dietary patterns identified in prior research [48–53]. In a systematic review and meta-analysis of 22 observational studies, patterns high in vegetables, fruits, whole grains, fish, and poultry were denoted “prudent/healthy” patterns [52]. In the multinational INTERHEART Study, a pattern rich in fruits and vegetables was also classified as “prudent” [50]. A “vegetarian-type” pattern characterized by higher consumption of vegetables, legumes, and boiled, baked, or mashed potatoes was identified in a case-control study conducted in Greece [48]. Specifically, within Mediterranean populations, diets abundant in plant-based foods, such as non-starchy vegetables, fruits, whole grains, legumes, olive oil, and nuts, alongside dairy products and/or fish and seafood, closely resembling the first component extracted in this study, have been deemed as representative of the traditional Mediterranean diet [49, 51, 53].

The Mediterranean diet variants are predominantly plant-based, featuring abundant vegetables, fruits, grains, legumes, nuts, and seeds, with olive oil as the main fat source; moderate consumption of mostly fermented dairy products; low-to-moderate fish and poultry consumption; minimal red meat; and wine, usually with meals [54, 55]. Robust scientific evidence from an umbrella review of meta-analyses indicates that adherence to this diet confers cardioprotection, reducing the risk of fatal and non-fatal CVD by 38%, CVD mortality by 41%, stroke by 36%, and myocardial infarction by 40% compared to other patterns [56]. Mechanistically, this pattern exerts anti-inflammatory, antioxidant and antithrombotic effects; improves lipid profiles; promotes endothelial function and vascular health; lowers arterial blood pressure; enhances insulin sensitivity [57]; and beneficially modulates gut microbiota composition [58]. The *traditional territorial Mediterranean diet*, acknowledged as “*intangible cultural heritage*” by UNESCO [59], reflects *a lifestyle pattern* that embodies the notions of frugality and eating in moderation and emphasizes the consumption of diverse seasonal, fresh or minimally processed, and locally produced foods [7, 55, 57]. Conviviality, the social dimension of eating, plays a key role in fostering interpersonal networks and social support. Beyond these social and ethical dimensions, this lifestyle involves skills, knowledge, and traditions in agriculture, culinary practices, and food preparation, with women playing a fundamental role in preserving these elements [59]. Additionally, regular moderate physical activity and sufficient sleep are integral components of this pattern [57]. In our study, participants with greater adherence to this pattern also exhibited lower smoking prevalence and reduced sedentary behavior. In terms of environmental sustainability, a modeling study estimated that adherence to the Mediterranean diet results in a carbon footprint of 2.3 kg CO_2_ eq/day/ca, aligning with planetary climate targets [60]. A systematic review reported GHG emissions of 0.9–6.9 kg CO_2_/day/ca, a water footprint of 600-5,280 m^3^/day/ca, and land use of 2.8–53.4 m^2^/day/ca [61]. Similarly, Naja et al. [62] found that the traditional Lebanese Mediterranean diet had the lowest water and carbon footprints per 1,000 kcal compared to Western and high-protein patterns. Furthermore, the cost of the Mediterranean diet is similar to other diets [61]. Furthermore, traditional variants support biodiversity conservation [55].

The Mediterranean dietary pattern served as the foundation for the EAT-LDP, which emphasizes plant-based foods, moderate fish and seafood intake, and minimal red meat and processed products to promote concurrently human health and planetary sustainability [10]. Several scoring systems have been developed to quantify adherence to the EAT-LDP, each employing distinct methodological approaches [17]. For example, in some scores, dietary components are assessed using ordinal scales [20], while other indices use continuous [16] or binary [22] scoring systems, affecting sensitivity and discriminatory ability [17]. A recent meta-analysis reported that greater adherence to EAT-LDP was associated with 16% reduced odds of major CVD, 17% lower CVD-specific mortality, and a 16% combined CVD risk reduction [17]. A similar cardioprotective relationship was found between EAT-LDP and 20-year fatal and non-fatal CVD risk in a recent publication from the ATTICA Study [18]. Prospective associations in the Malmö Diet and Cancer Study unraveled that participants in the highest adherence group experienced a 32% reduction in CVD mortality compared to the lowest adherence group after an average follow-up of 20 years [20]. Further long-term analyses of this population-based study revealed that higher levels of adherence were linked to lower risks of atrial fibrillation [19], heart failure [25], and coronary heart disease (CHD) [23]. Additionally, in British [22] and Dutch cohorts [24], high adherence was associated with 28% and 15% reductions in CHD risk after 23.6 and 15.3 years, respectively. Findings from a Danish cohort demonstrated an inverse association between EAT-LDP adherence and risk of subarachnoid hemorrhage over a median observational period of 15 years [21]. In a pooled US sample of > 200,000 participants, those in the highest adherence category exhibited a 17% lower CVD incidence, 19% decreased CHD, 14% reduced stroke risk [27], and 14% reduced CVD-specific mortality [30]. Consistent inverse associations between EAT-LDP adherence and CVD outcomes were observed in other US [28, 29, 33], UK [31, 32], and Iranian [34] cohorts. However, other investigators reported no statistically significant outcomes when using scores based on EAT-LDP [15, 26] or the SDI [35]. In literature, high adherence to EAT-LDP has been correlated with a 2.4% decrease in GHG emissions, 3.9% lower land footprint, 0.5% reduced freshwater eutrophication, 3.3% less marine eutrophication, and 7.7% less terrestrial acidification, albeit with a 32.1% increase in blue water consumption [16].

The second dietary pattern derived from our analyses, defined as “Western animal-based, processed food pattern,” corresponds to patterns high in red and/or processed meat, fried potatoes, refined grains, sweets, or fast foods, which have been identified as “Western” [48, 49, 52, 63] or “unhealthy” [52] patterns in other studies. Meta-analytic data link the highest adherence to a 14% greater CVD and 3% greater CHD risk [52]. Evidence from a case-control study linked adherence to such a diet with 20% greater odds of CHD [48]. In a female cohort, the highest quintile was correlated with 1.58- and 1.56-times higher risk of total and ischemic stroke, respectively [63]. Nonetheless, two meta-analyses [64, 65], two prospective studies [66, 67], and a cross-sectional study in Greece [49] failed to find significant associations between Western-style patterns and CVD outcomes, which is consistent with our findings. These discrepancies have been attributed to methodological limitations inherent to *a posteriori* dietary pattern analyses or population-specific dietary heterogeneity [49, 66]. Overall, dietary shifts toward Western-type diets induce chronic low-grade inflammation, oxidative stress, changes in mitochondrial processes, dyslipidemia, and insulin resistance, while also promoting gut microbiota dysbiosis and intestinal barrier dysfunction [68], thereby increasing susceptibility to diet-related non-communicable diseases, including CVD. Environmentally, Western dietary patterns impose substantially higher resource demands and carbon footprints than Mediterranean diets [61]. For example, approximately one-third of arable land is occupied for cultivation of animal feed for livestock, representing a primary driver of deforestation and biodiversity loss [10, 69]. Additionally, ruminants emit enteric methane, a potent GHG with a global warming potential 28-fold greater than that of carbon dioxide over a 100-year timescale [70]. These impacts exacerbate climate change, accelerate ecosystem degradation, and contribute to food insecurity, thereby triggering social instability and undermining social equity [8]. Moreover, the globalization of Western diets has detrimental effects on traditional cultural backgrounds [71]. Economically, the Western diet imposes considerable costs on healthcare systems [68].

The third component represents a hypercaloric dietary pattern exceeding metabolic requirements with low white meat consumption. As seen in Table 1, it is marked by lower intake of nutrient-dense foods, such as vegetables and fruits, alongside a positive association with sweets and confectionery, demonstrating a higher proportion of energy intake from added sugars rather than from plant-based sources. Therefore, this pattern diverges from recommendations to shift energy away from animal-sourced and processed foods and toward plant-derived foods [7]. Additionally, participants with higher adherence to this dietary pattern also had less optimal body weight and anthropometric indices profiles.

### Policy measures to achieve health and sustainability in food consumption

Contemporary adherence to the traditional territorial dietary patterns, such as the Mediterranean diet, has declined [72], a trend mirrored by the rising per-capita carbon footprint of food consumption in Mediterranean countries [60]. Moreover, most national food-based dietary guidelines (FBDG) fail to align with global health and environmental targets [60]. Therefore, a shift towards healthier and more sustainable dietary patterns is an effective strategy to contribute to global Public Health and emission reduction targets [7, 8]. This approach is central to the European Union’s Farm to Fork strategy, which is integral to the European Green Deal’s objective of achieving carbon neutrality by 2050 [11]. Although vegan and vegetarian diets exhibit the lowest environmental footprints, large-scale shifting towards these patterns, which seems unrealistic, may pose challenges for nutritional adequacy, requiring substantial nutrition literacy and behavioral discipline to prevent nutrient deficiencies if not carefully planned [73]. Hence, advancing this shift necessitates a multidimensional, holistic approach, including the revision of the national FBGD and the implementation of Public Health campaigns, with the aim to promote traditional territorial dietary patterns, such as the Mediterranean diet, across the pillars of health, environmental, social, and economic sustainability.

Strengths and limitations.

The ATTICA Study features a prospective design with multiple follow-ups over a 20-year period, enabling a comprehensive assessment of the association between dietary habits and long-term CVD outcomes. However, limitations should be acknowledged. Self-reported FFQ dietary intake may be prone to measurement errors and recall bias. Nevertheless, this FFQ had been previously validated in the Greek population and was administered by trained dietitians. Moreover, dietary data collection was performed continuously over an entire year, thereby capturing dietary habits from all seasons. Furthermore, although PCA effectively identifies prevailing dietary patterns within a cohort, it has some limitations, such as that the extracted components remain subject to researcher interpretation. Some attrition-related selection bias also cannot be excluded, as participants lost to follow-up had a less favorable socio-economic profile.

## Conclusion

In the Anthropocene epoch, a high-emission and resource-intensive era, the growing CVD burden calls for integrated prevention strategies that prioritize both health and sustainability. Our findings demonstrate that adherence to a plant-based, traditional, territorial dietary pattern, such as the Mediterranean diet, can reduce long-term CVD risk while supporting environmental goals. Effective policy measures should foster sustainable dietary transitions through revised FBDG and Public Health initiatives aligned with global climate and health objectives, with primordial and primary prevention emphasized as a priority.

## Supplementary Information

Below is the link to the electronic supplementary material.

Supplementary Material 1

## Data Availability

Data described in the manuscript, code book, and analytic code will be made available upon request to the corresponding author.
