# Supplementary material for: Sustainable diets and long-term cardiovascular disease outcomes; insights from the 20-year follow-up ATTICA study (2002–2022)
Source: Eur J Nutr. 2026 Jun 19;65(5):169. doi: 10.1007/s00394-026-04022-7 (PMC13282287; doi:10.1007/s00394-026-04022-7)
Supplement: Supplementary file 1 — Supplementary Material 1 [file 394_2026_4022_MOESM1_ESM.docx]

| **Table 1S.** Results from nested Cox proportional hazards models assessing the relationship between tertiles of adherence to the three orthogonal dietary patterns and 20-year CVD incidence among participants of the ATTICA Study (*n=* 1,988). | | | | | | |  |
| --- | --- | --- | --- | --- | --- | --- | --- |
|  | **Tertiles of plant-based, sustainable dietary pattern** | | **Tertiles of western animal-based, processed food pattern** | | **Tertiles of high-calorie, low-white-meat dietary pattern** | | |
|  |  | ***p* for trend** |  | ***p* for trend** |  | ***p* for trend** | |
| **Unadjusted model** | T1: 1.00 reference | 0.450 | T1: 1.00 reference | *<0.001* | T1: 1.00 reference | 0.458 | |
|  | T2: 1.19 (0.65, 2.18) |  | T2: 0.36 (0.20, 0.68) ^**^ |  | T2: 0.41 (0.22, 0.79) ^**^ |  | |
|  | T3: 0.80 (0.42, 1.51) |  | T3: 0.38 (0.20, 0.72) ^**^ |  | T3: 0.65 (0.36, 1.20) |  | |
|  |  | ***p* for trend** |  | ***p* for trend** |  | ***p* for trend** | |
| **Adjusted model** | T1: 1.00 reference | 0.559 | T1: 1.00 reference | 0.415 | T1: 1.00 reference | 0.520 | |
|  | T2: 0.72 (0.30, 1.69) |  | T2: 0.61 (0.26, 1.44) |  | T2: 0.41 (0.22, 0.79) |  | |
|  | T3: 0.44 (0.18, 1.10) |  | T3: 0.74 (0.31, 1.78) |  | T3: 0.65 (0.36, 1.20) |  | |
|  |  | ***p* for trend** |  | ***p* for trend** |  | ***p* for trend** | |
| **Fully adjusted model** | T1: 1.00 reference | *0.047* | T1: 1.00 reference | 0.423 | T1: 1.00 reference | 0.518 | |
|  | T2: 0.65 (0.27, 1.57) |  | T2: 0.59 (0.25, 1.40) |  | T2: 0.43 (0.18, 1.03) |  | |
|  | T3: 0.39 (0.15, 0.98) ^*^ |  | T3: 0.73 (0.31, 1.76) |  | T3: 0.82 (0.34, 2.00) |  |  |
| Results are presented as HR (95% CI).  Adjusted model: Age + Sex + Socio-economic status + Hypertension + Hypercholesterolemia + Diabetes mellitus + WHR  Fully adjusted model: further adjusted for Smoking exposure + Sedentary lifestyle status  ^***^ *p*< 0.001, ^**^ *p*< 0.01, ^*^ *p*< 0.05  *Abbreviations*: HR: hazards ratio, Ti: i^th^ tertile, WHR: waist-to-hip ratio, 95% CI: 95% confidence interval. | | | | | | |  |

| **Table 2S.** Results from statistical models assessing the relationship between tertiles of adherence to the three orthogonal dietary patterns and CVD-related lifetime risk and DALYs among participants of the ATTICA Study (*n=* 1,988). | | | | | | |  |
| --- | --- | --- | --- | --- | --- | --- | --- |
|  | **Tertiles of plant-based, sustainable dietary pattern** | | **Tertiles of western animal-based, processed food pattern** | | **Tertiles of high-calorie, low-white-meat dietary pattern** | | |
|  |  | ***p* for trend** |  | ***p* for trend** |  | ***p* for trend** | |
| **Lifetime risk** | T1: 1.00 reference | 0.378 | T1: 1.00 reference | 0.298 | T1: 1.00 reference | *0.009* | |
|  | T2: -0.026 ± 0.048 |  | T2: -0.059 ± 0.049 |  | T2: 0.077 ± 0.048 |  | |
|  | T3: -0.044 ± 0.049 |  | T3: -0.053 ± 0.050 |  | T3: 0.128 ± 0.049 ^**^ |  | |
| **DALYs** |  | ***p* for trend** |  | ***p* for trend** |  | ***p* for trend** | |
|  | T1: 1.00 reference | 0.968 | T1: 1.00 reference | 0.464 | T1: 1.00 reference | *0.025* | |
|  | T2: -0.067 ± 0.128 |  | T2: -0.133 ± 0.124 |  | T2: 0.43 ± 0.13 ^**^ |  | |
|  | T3: -0.009 ± 0.137 |  | T3: -0.076 ± 0.130 |  | T3: 0.28 ± 0.12 ^*^ |  | |
| Results are presented as β ± SE. Fully adjusted models were  adjusted for: Age + Sex + Socio-economic status + Hypertension + Hypercholesterolemia + Diabetes mellitus + WHR + Smoking exposure + Sedentary lifestyle status  ^***^ *p*< 0.001, ^**^ *p*< 0.01, ^*^ *p*< 0.05  *Abbreviations*: DALYs: disability-adjusted life years, SE: standard errors, Ti: i^th^ tertile, WHR: waist-to-hip ratio, 95% CI: 95% confidence interval. | | | | | | |  |
